# Fenugreek Seed Extract Inhibit Fat Accumulation and Ameliorates Dyslipidemia in High Fat Diet-Induced Obese Rats

**DOI:** 10.1155/2014/606021

**Published:** 2014-04-29

**Authors:** Parveen Kumar, Uma Bhandari, Shrirang Jamadagni

**Affiliations:** ^1^Department of Pharmacology, Faculty of Pharmacy, Hamdard University, New Delhi 110062, India; ^2^Department of Pharmacology, National Research Institute of Ayurvedic Drug Development, Kolkata 700091, India

## Abstract

This study investigated the inhibitory effect of aqueous extract of *Trigonella foenum-graecum* seeds (AqE-TFG) on fat accumulation and dyslipidemia in high fat diet- (HFD-) induced obese rats. Female Wistar rats were fed with HFD *ad libitum*, and the rats on HFD were treated orally with AqE-TFG or orlistat ((HFD for 28 days + AqE-TFG (0.5 and 1.0 g/kg) or orlistat (10 mg/kg) from day 8 to 28), respectively. Treatment with AqE-TFG produced significant reduction in body weight gain, body mass index (BMI), white adipose tissue (WAT) weights, blood glucose, serum insulin, lipids, leptin, lipase, and apolipoprotein-B levels and elevation in adiponectin levels. AqE-TFG improved serum aspartate amino transferase (AST), alanine amino transferase (ALT), and lactate dehydrogenase (LDH) levels. AqE-TFG treatment reduced the hepatic and cardiac thiobarbituric acid reactive substances (TBARS) and elevated the antioxidant enzyme (glutathione (GSH), superoxide dismutase (SOD), and catalase (CAT)) levels. In addition, liver and uterine WAT lipogenic enzyme (fatty acid synthetase (FAS) and glucose-6-phosphate dehydrogenase (G6PD)) activities were restored towards normal levels. These findings demonstrated the preventive effect of AqE-TFG on fat accumulation and dyslipidemia, due to inhibition of impaired lipid digestion and absorption, in addition to improvement in glucose and lipid metabolism, enhancement of insulin sensitivity, increased antioxidant defense, and downregulation of lipogenic enzymes.

## 1. Introduction

Obesity is a chronic disorder of carbohydrate and lipid metabolism and is characterized by an increased fat deposition in adipose tissue and other internal organs [1]. Obesity leads to the development of insulin resistance, type-2 diabetes, coronary heart disease, cancer, respiratory disease, and osteoarthritis [2]. During the past 3-4 decades, changes in the food system seem to be the major drivers of the rise of the global epidemic of obesity [3]. As per the World Health Organization (WHO), in 2008, more than 1.4 billion adults (20 years of age and older) were overweight and amongst these over 200 million men and nearly 300 million women were obese [4].

Over the years, many medications have been used to manage obesity but most of them are now withdrawn due to their serious adverse effects [5]. Currently, orlistat is the only Food and Drug Administration (FDA) approved drug for long term management of obesity but this drug has undesirable gastrointestinal side effect such as steatorrhea [6]. In the face of this unmet medical need, there is requirement of new potential antiobesity drug to combat this syndrome.

Fenugreek (*Trigonella foenum-graecum* L.; TFG) belongs to the family Fabaceae and is used in many parts of the world for the treatment of diabetes. TFG seeds are used as an active ingredient in weight loss and anticholesterol ayurvedic formulation Ayurslim (The Himalaya Drug Company, Bangalore, India). TFG seeds have been shown to possess hypoglycemic, hypolipidemic, and antioxidant effects [7].

Dietary supplemented protein and amino acids have been shown to reduce plasma lipid levels [8, 9]. The presence of proteins and fiber in TFG seeds offers high nutritive value as it contains approximately 26% protein and 48% fiber and might exert a lipid lowering effect [10]. Dietary fibers (galactomannan) in fenugreek seeds are polysaccharides consisting of a mannose backbone with galactose side chains attached at position C6. Galactomannan consist of linear chains of (1–4) diequatorially linked D-mannose residues; some contain single-sugar side chains of D-galactose attached by (1–6) glycosidic bonds [11]. Fenugreek galactomannan form a viscous gel in the intestine and inhibit glucose and lipid absorption [12].

Oxidative stress is a biochemical disequilibrium occurring due to enhanced generation of reactive oxygen species (ROS). Elevated oxidative stress could be responsible for the initiation of metabolic disorders due to alterations in the membrane lipids and proteins [13]. In epidemiological studies, polyphenol and flavonoids-rich extract have been shown to possess the hypocholesterolaemic effect due to their antioxidant defense [14].

As plant proteins, dietary fiber and polyphenols do possess health benefits in metabolic disorders; therefore, in the present study, the inhibitory effect fenugreek extract was evaluated on fat accumulation and dyslipidemia in high fat diet-induced obese rats.

## 2. Materials and Methods

### 2.1. Plant Materials and Preparation of Extract

*Trigonella foenum-graecum* seeds were purchased from Indian Council for Agricultural Research (ICAR), New Delhi, India (ref. NISCAIR/RHMD/Consult/-2011-12/1743/43). The seeds were dried at 40°C and ground to a powder. The aqueous extract of* Trigonella foenum-graecum* seeds (AqE-TFG) was prepared as follows: powdered seeds were soaked in hot distilled water (1 : 20 w/v) and left overnight. The solution was filtered and lyophilized.

### 2.2. Electrophoretic Fingerprinting of Proteins in AqE-TFG

AqE-TFG was dissolved in phosphate buffer saline (PBS; pH 7.4; 1 : 10 w/v) and centrifuged (15,000** ×**g, 30 min). The clear supernatant was dialyzed against PBS at 4°C (5000 cutoff membrane; with fresh buffer changes every 6 h, 4 times). Sodium dodecyl sulfate-polyacrylamide gel electrophoresis (SDS-PAGE) analysis was carried out as described by Laemmli [15], using 12% resolving gel and 5% stacking gel. Protein bands were stained with Coomassie Brilliant Blue R-250 in methanol-acetic acid-water (5 : 1 : 5 v/v/v) and decolorized in 7% acetic acid. Molecular weight of different bands was calibrated with the standard proteins markers: phosphorylase b (molecular weight, 97.4 kDa), albumin (66.2 kDa), ovalbumin (45 kDa), carbonic anhydrase (29 kDa), trypsin inhibitor (20.1 kDa), and *α*-lactalbumin (14.4 kDa).

### 2.3. Amino Acid Analysis from AqE-TFG

The precolumn derivatisation of amino acid in AqE-TFG was performed with 6-aminoquinolyl-N-hydroxyl succinimidyl carbamate (AQC) and the concentration of amino acid was analysed by reverse phase high performance liquid chromatography (HPLC) (Waters Co., Milford, MA, USA). The excitation and emission wavelengths for fluorescence detector were 250 nm and 395 nm, respectively. The gain setting of the detector was 1, and the 5 *μ*L of samples was injected onto Inertsil ODS-3V column (250 × 4.6 mm, 4 *μ*m) with the temperature controlled at 37°C and operated with a flow rate of 1.0 mL/min. The linear gradient elution system was used. Mobile phases A, B, and C were acetate buffer (pH 5.05) solution, acetonitrile, and water, respectively. The linear gradient conditions of solvents were modified as follows: initial, 100% A (acetate buffer); 0.5 min, 99% A, 1% B (HPLC grade acetonitrile); 18 min, 95% A, 5% B; 29.5 min, 83% A, 17% B; 33 min, 60% B, 40% C (Milli-Q water); 36 min, 100% A; 65 min, 60% B, 40% C; and 100 min, 60% B, 40% C.

### 2.4. Determination of Galactomannan in AqE-TFG

AqE-TFG was solubilized in water (1 : 20, w/v) at 4°C for 3 hours and centrifuged (16,000 ×g, 30 min). The supernatant was mixed with absolute ethanol (1 : 1, v/v) to precipitate galactomannan [12].

### 2.5. Determination of Phenolic and Flavonoid Contents in AqE-TFG

Phenolic contents of the extract were determined as follows: 50 *μ*L aliquot of extract was assayed with 250 *μ*L of Folin-Ciocalteu reagent and 500 *μ*L of sodium carbonate (20%, w/v). The mixture was vortexed and diluted with water to a final volume of 5 mL. After incubation for 30 min at room temperature, the absorbance was read at 765 nm [16].

Flavonoid contents of the extract were determined as follows: 0.5 mL of sample; 0.5 mL of 2% AlCl_3_ (w/v) in 95% ethanol solution was added. After 1 h at room temperature, the absorbance was measured at 420 nm [17].

### 2.6. Experimental Design

The guidelines of Committee for the Purpose of Control and Supervision of Experiments on Animals (CPCSEA), Government of India, were followed and prior permission was sought from the Institutional Animal Ethics Committee (IAEC), Hamdard University (registration number 173/CPCSEA; protocol number 773), New Delhi, India, for conducting the study.

The female Wistar rats (150–200 g) were housed in polypropylene cages under controlled conditions (room temperature 25 ± 2°C, air humidity 50 ± 15%, and photoperiod of 12 h light: dark cycle), and water was provided* ad libitum* throughout the study. After acclimatization to the environment for one week, the animals were randomly divided into 5 groups (*n* = 10 rats in each group). Normal control rats (Group 1) were fed with normal pellet diet (NPD), while the others (Groups 2, 3, 4, and 5) were fed with high fat diet (HFD)* ad libitum* for 28 days. From day 8 to 28, Groups 3 and 4 were treated with AqE-TFG (0.5 and 1.0 g/kg body weight (b.w.), orally) while Group 5 was treated with orlistat (10 mg/kg b.w., orally). The solutions of AqE-TFG and orlistat were freshly prepared using normal saline. The compositions of NPD (Amrut rat feed, Mfd. by Nav Maharashtra Chakan Oil Mills Ltd., Delhi, India) and HFD (National Institute of Nutrition, Hyderabad, India) are shown in Table 1.

### 2.7. Collection of Serum and Tissues

On day 29, blood was collected from the retroorbital plexus of overnight fasted rats under light ether anaesthesia and serum was separated by centrifugation at 3000 rpm for 15 min. Then, the rats were sacrificed. The organs (heart and liver) and white adipose tissue (WAT) (mesenteric, gonadal, and retroperitoneal) were excised, rinsed in ice-cold physiological saline, and weighed. The serum and organ samples were stored at −20°C until analysis.

### 2.8. Determination of Anthropometric Parameters

Body weight gain was calculated as a difference of initial and final weight of animals. Body mass index (BMI) was calculated from the formula BMI = body weight (Kg)/length^2^ (m^2^) [18]. Food and water intakes were measured daily for a period of 28 days and the average of food and water consumption was calculated. Blood pressure (systolic blood pressure (SBP), diastolic blood pressure (DBP), and mean arterial pressure (MAP)) and heart rate (HR) were measured with a noninvasive blood pressure recorder using the rat tail-cuff method (Kent Scientific Corporation).

### 2.9. Determination of Serum Biochemistries

The blood glucose, serum triglycerides (TGs), total cholesterol (TC), high density lipoprotein cholesterol (HDL-C) concentrations, aspartate amino transferase (AST), and alanine amino transferase (ALT) activities were assayed by enzymatic methods using commercial assay kits (Span Diagnostics Ltd., Surat, India) according to the manufacturer's protocol. The serum lactate dehydrogenase (LDH) concentration was determined using an enzymatic kit (Reckon Diagnostics Pvt. Ltd., Baroda, India). Rat insulin immunoassay ELISA kit (Crystal Chem, Inc., IL, USA), rat leptin immunoassay ELISA kit (Ray Biotech Inc., GA, USA), rat adiponectin EIA kit (Ray Biotech Inc., GA, USA), apo-B immunoturbidimetric immunoassay kit (Randox Laboratories Ltd., UK), and lipase kit (BioAssay Systems, CA, USA) were used to measure the serum insulin, leptin, adiponectin, apo-B, and lipase concentrations.

Low density lipoprotein cholesterol (LDL-C) and very low density lipoprotein cholesterol (VLDL-C) levels were estimated using Friedewald's equation: LDL-C=TC−HDL-C−VLDL-C; VLDL-C = TGs/5 [19]. The atherogenic index (AI) and coronary risk index (CRI) were calculated as log (TG/HDL-C) [20] and TC/HDL-C [21], respectively.

Homeostasis model assessment for insulin resistance (HOMA-IR) and quantitative insulin sensitivity check index (QUICKI) were calculated as fasting glucose (mg/dL) × fasting insulin (*μ*U/mL)/2430 [22] and 1/[log insulin (*μ*U/mL) + log glucose (mg/dL)] [23], respectively.

### 2.10. Determination of Tissue Biochemistries

The thiobarbituric acid reactive substances (TBARS) [24] and antioxidant enzymes activities, that is, reduced glutathione [25], superoxide dismutase (SOD) [26], and catalase (CAT) [27], were measured in hepatic and cardiac tissues. The fatty acid synthase (FAS) [28] and glucose-6-phosphate dehydrogenase (G6PD) [29] activities were assayed in hepatic and uterine WAT.

### 2.11. Histopathological Analysis

For histological examination, the liver tissue was collected, fixed in 10% neutral buffered formalin, and embedded in paraffin. Standard sections of 5 *μ*m thickness were cut and stained with hematoxylin and eosin (H & E). The slides were examined by light microscopy.

### 2.12. Statistical Analysis

The data are expressed as means ± S.E.M. All statistical analyses were performed using Graph-Pad InStat version 3.06 (Graph Pad Software). All data were analyzed using 1-way analysis of variance (ANOVA) followed by Dunnett's test. Results were considered statistically significant when *P* < 0.05.

## 3. Results

### 3.1. Chemical Compositions of AqE-TFG

**Figure 1:**
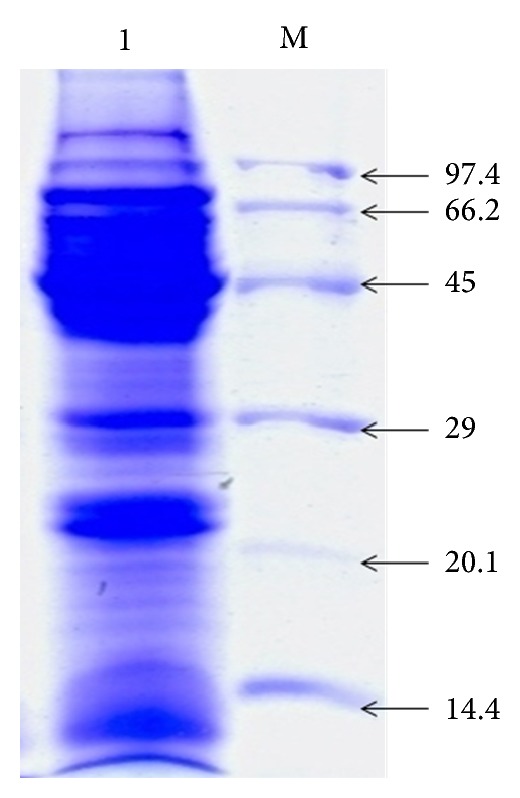
Electrophoretic profile of aqueous extract of* Trigonella foenum-graecum* (AqE-TFG) proteins. Lane 1 indicates AqE-TFG proteins of molecular weight from 14.4 kDa to 97.4 kDa wherein thick protein bands lies <66.2 kDa; Lane M indicates standard molecular weight markers (kDa).

The electrophoretic fingerprinting of AqE-TFG revealed that AqE-TFG contains proteins of molecular weight from 14.4 kDA to 97.4 kDA wherein thick protein bands lie <66.2 kDA (Figure 1). The contents of amino acid, phenolic, flavonoid, and galactomannan in AqE-TFG are summarized in Table 2.

### 3.2. Effect of AqE-TFG on Anthropometric Parameters

The feeding of HFD for 28 days caused a significant (*P* < 0.01) increase in body weight gain and BMI of rats, in comparison with the NPD control rats. Treatment with AqE-TFG (0.5 and 1.0 g/kg) or orlistat (10 mg/kg) for 21 days significantly (*P* < 0.05 or *P* < 0.01) suppressed the increase in the body weight gain and BMI of HFD-fed rats. Despite variation in body weight gain and BMI, there was no significant difference in food intake and water intake amongst all groups (Table 3).

A statistically significant (*P* < 0.01) increase in SBP, DBP, and MAP was observed in the HFD control group than those in NPD group. AqE-TFG (0.5 and 1.0 g/kg) or orlistat (10 mg/kg) treatment significantly (*P* < 0.01) decreased the elevated SBP, DBP, and MAP in HFD-fed rats (Table 4).

The weights of WAT (mesenteric, gonadal, and retroperitoneal) and organ (heart and liver) were significantly (*P* < 0.01) higher in HFD control group than those in NPD group. AqE-TFG (0.5 and 1.0 g/kg) or orlistat (10 mg/kg) treatment significantly (*P* < 0.05 or *P* < 0.01) lowered the WAT as well as organ weights when compared to the HFD control group (Table 5).

### 3.3. Effect of AqE-TFG on Serum Biochemistries

Table 6 represents the lipid profile of experimental rats. Rats in HFD control group displayed a significant (*P* < 0.01) increase in the levels of TC, TGs, LDL-C, and VLDL-C and a significant (*P* < 0.01) decrease in HDL-C in comparison with NPD group. Similar with the orlistat-treated HFD-fed group, AqE-TFG (0.5 and 1.0 g/kg) administration showed a significant (*P* < 0.01) decrease in the levels of serums TC, TGs, LDL-C, and VLDL-C or a significant increase in the level of HDL-C after 21-day treatment when compared with HFD control group. The cardiac risk indexes (AI and CRI) were significantly (*P* < 0.01) increased in HFD control group when compared to NPD group, while treatment with AqE-TFG (0.5 and 1.0 g/kg) or orlistat (10 mg/kg) caused a significant reduction in the AI and CRI levels compared to HFD control group.

The HFD-induced obese rats exhibited a significant (*P* < 0.01) increase in the levels of blood glucose, serum insulin, leptin, apo-B, and HOMA-IR as compared to NPD-fed rats. After AqE-TFG (0.5 and 1.0 g/kg) or orlistat (10 mg/kg) treatment for 21 days, the increases of blood glucose, serum insulin, leptin, apo-B, and HOMA-IR were significantly brought down towards normal in HFD-fed rats (Table 7).

A statistically significant (*P* < 0.01) decrease in adiponectin levels and QUICKI was observed in the HFD control group than those in NPD group. AqE-TFG (0.5 and 1.0 g/kg) or orlistat (10 mg/kg) treatment significantly (*P* < 0.01) elevated the reduced levels of adiponectin and QUICKI compared to HFD control group (Table 7).

The serum lipase, LDH, AST, and ALT levels were significantly (*P* < 0.01) increased in HFD control group, when compared to the NPD group. A significant (*P* < 0.01) reduction in lipase, LDH, AST, and ALT was observed in AqE-TFG (0.5 and 1.0 g/kg) or orlistat (10 mg/kg) treated group in comparison to the HFD control group (Table 8).

### 3.4. Determination of Tissue Biochemistries

Table 9 represents the lipogenic enzyme activities of the hepatic and uterine WAT. The HFD control group showed a significant (*P* < 0.01) elevation in levels of lipogenic enzyme (FAS and G6PD) as compared to the NPD control group. Treatment with AqE-TFG (0.5 and 1.0 g/kg) or orlistat (10 mg/kg) for a period of 21 days caused a significant (*P* < 0.01) reduction in levels of FAS and G6PD, respectively, as compared to the HFD control group.

Table 10 represents the oxidative stress marker in hepatic and cardiac tissue. A marked increase of TBARS production and decrease of antioxidant enzyme status (GSH, SOD, and CAT) were observed in the hepatic and cardiac tissue of rats in HFD control group when compared with NPD group. AqE-TFG or orlistat treatment significantly (*P* < 0.01) decreases the TBARS levels and raises the antioxidant enzyme (GSH, SOD, and CAT) activity.

### 3.5. Hepatic Histopathology

**Figure 2:**
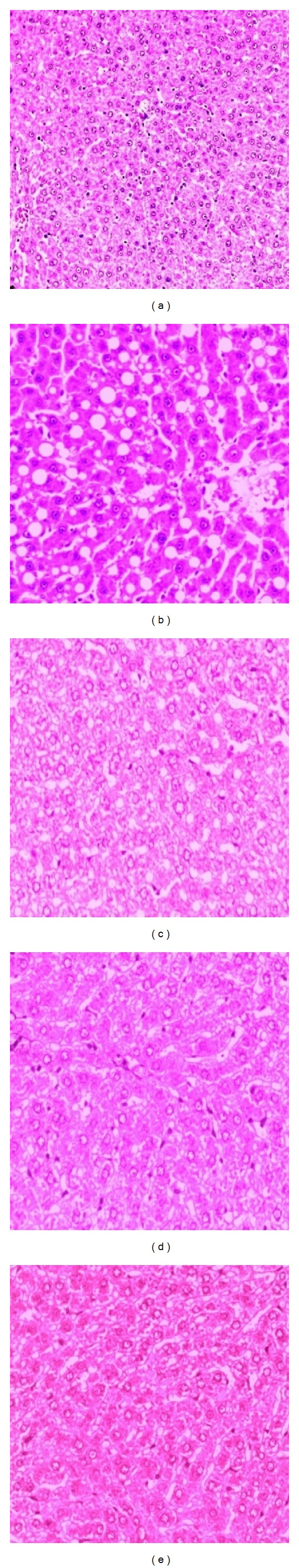
Effect of aqueous extract of* Trigonella foenum-graecum* (AqE-TFG) on hepatic histopathological changes in high fat diet- (HFD-) induced obese rats. Group 1 (a): NPD control, showing normal liver architecture and hepatocytes; Group 2 (b): HFD control, showing fatty degeneration and greater hepatic lipid accumulation; Group 3 (c): HFD + AqE-TFG (0.5 g/kg, b.w.), showing mild congestion, no fatty changes, and less hepatic lipid accumulation; Group 4 (d): HFD + AqE-TFG (1.0 g/kg, b.w.), showing no fatty changes and considerably lower hepatic lipid accumulation; Group 5 (e): HFD + orlistat (10 mg/kg, b.w.), showing no fatty changes with lower hepatic lipid accumulation.

Liver histopathologies are shown in Figure 2. The histopathological examination of the NPD control group showed normal cell architecture, while HFD control group showed significant morphological changes with greater hepatic lipid accumulation and fatty degeneration as compared to NPD control rats. On the other hand, treatment with AqE-TFG (0.5 and 1.0 g/kg) or orlistat (10 mg/kg) for a period of 21 days in HFD-fed rats lowered hepatic lipid accumulation as well as fatty degeneration as compared to HFD control rats, respectively.

## 4. Discussion

In the present study, consumption of high caloric intake in form of HFD for a period of 28 days induces obesity in rats. BMI is widely used to measure body fat and highly correlated with body fat stores [30]. In our study, a significant reduction in body weight gain and BMI with AqE-TFG treatment indicates that AqE-TFG suppresses the HFD-mediated increase in body weight gain and WAT weight. Despite a significant difference in body weight between the NPD control and HFD control groups, there was no significant difference in the food intake and water intake.

Leptin is a hormone secreted from adipose tissue and regulates appetite and adiposity. With the increase in adipose tissue weight, serum leptin levels also tend to increase [31]. In the present study, the reduction of leptin levels indicated that AqE-TFG treatment caused significant adipocytes loss. This observation was further supported by the AqE-TFG mediated reductions in WAT weights and adiposity index (sum of the weight of the mesenteric, gonadal and retroperitoneal WAT, divided by the weight of the animal, and expressed as percentage) of HFD-fed rats.

Obesity is an independent risk factor for cardiovascular disease (CVD), through its influence on other known risk factors such as dyslipidemia and hypertension [32]. Apo-B is synthesized in liver and indicates the amount of atherogenic lipoproteins in plasma or hepatic tissue, and it is considered as a better predictor of coronary heart disease [33]. In our study, administration of AqE-TFG caused a significant reduction in serum lipids (TC, TGs, LDL-C, and VLDL-C), cardiac risk indexes (AI and CRI), and apo-B levels which may be considered as a better indicator for improvement in risk of coronary heart disease. Further, it is well established that dyslipidemia and hypertension are the risk factors for coronary heart disease [34]. Therefore, cardiovascular risk can be minimised through regulation of dyslipidemia and hypertension. The reduction in the SBP, DBP, and MAP with AqE-TFG indicates that AqE-TFG may be a potential candidate in management of dyslipidemia-induced cardiac complications.

With the increase in adipose tissue fat deposits, as in obesity, the ability of insulin to stimulate glucose transport and metabolism in adipocytes and skeleton muscle is impaired resulting in insulin resistance [35]. Impairment in insulin sensitivity led to dyslipidemia [36]. Further, small adipocytes are more sensitive to insulin than the large adipocytes [37]. The AqE-TFG mediated reduction in HOMA-IR and elevation in QUICKI indicated the improvement in insulin sensitivity, which is due to depletion of the adipose tissue triglycerides stores that ultimately results in reductions of lipid levels.

Adiponectin (an adipose-tissue-derived hormone) plays an important role in the regulation of lipid metabolism and insulin sensitivity and also possesses anti-inflammatory and antiatherogenic properties. Adiponectin exhibits insulin sensitizing effect, in part via AMPK activation in peripheral tissues that led to stimulation of fatty acid oxidation and glucose uptake in skeletal muscle, and suppression of glucose production in liver. Circulating adiponectin levels are negatively correlated with obesity, particularly visceral obesity and insulin resistance [38]. In the present study, higher adiponectin levels with AqE-TFG treatment indicated a protective role in the development of metabolic disorders.

The feeding of high fat diet results in excess hepatic triglycerides accumulation due to increased synthesis and decreased secretion of triglycerides and increased de novo lipogenesis [39]. Dietary lipids cannot absorb from the intestinal linings without undergoing hydrolysis by lipase. Inhibition of lipase activity leads to decrease in intestinal lipid digestion and absorption [12]. In the present study, similar to orlistat treated group, the reduced lipase activity with AqE-TFG treatment indicated an inhibitory effect on intestinal dietary fat absorption that leads to decreased triglycerides accumulation in various tissue including liver and WAT. This finding was further supported by the hepatic histopathological studies that showed reductions in hepatic lipid accumulation, fatty degeneration with AqE-TFG, and orlistat treatment.

It is reported that the levels of hepatic and cardiac markers such as ALT, AST, and LDH tend to increase in obesity [40]. The elevated levels of these enzymes in our study indicated that HFD control rats are prone to hepatic and cardiac complications. Treatment with AqE-TFG for a period of 21 days significantly reduced the increased levels of LDH, AST, and ALT.

Factors like enzymes, nutritional conditions, and hormones control the process of fat synthesis and fat breakdown [41]. Elevated lipogenesis is strongly associated with obesity, fatty liver disease, insulin resistance, and type-2 diabetes [42]. Fatty acid synthase (FAS) is responsible for the synthesis of long-chain fatty acids from acetyl-CoA, malonyl-CoA, and NADPH [43]. Enzymes of the pentose phosphate pathway including glucose-6-phosphate dehydrogenase (G6PD) and 6-phosphogluconate dehydrogenase (6PGD) produce NADPH which is essential for the biosynthesis of fatty acid and cholesterol [44]. Therefore, modulation of the activities of liver and epididymal WAT lipogenic enzyme (FAS and G6PD) with AqE-TFG treatment indicated the reduced availability of NADPH required for the biosynthesis of fatty acid and cholesterol, leading to rapid decline in fat stores of HFD-fed rats.

Abnormalities in lipid metabolism decrease the strength of the antioxidative defenses [45]. The correlation found between dyslipidemia and oxidative stress in this study shows that dyslipidemia induced by ingestion of high fat diet is the primary cause of lipid peroxidation. Therefore, the possible reason for improvement in dyslipidemia with AqE-TFG may be due to reduction in oxidative stress in HFD fed rats. In the present study, HFD-induced dyslipidemic rats showed decreased activities of GSH, SOD, and CAT enzymes; therefore, it may be concluded that HFD causes the induction of oxidative stress in the hepatic and cardiac tissue and may lead to the consequences like fatty liver disease and atherosclerosis. Administration of AqE-TFG for a period of 21 days resulted in significant reduction in lipid peroxides levels and elevation in antioxidant enzymes GSH, SOD, and CAT.

Earlier, proteins, galactomannan, and polyphenols from fenugreek seeds have been reported to regulate dyslipidemia in obese and diabetic rodents [12, 14, 46]. Therefore, in our study, the presence of these phytoconstituents justified the fact that AqE-TFG effectively inhibit fat accumulation and ameliorate dyslipidemia in HFD-obese rats, which is due to prevention of impaired lipid digestion and absorption, in addition to improvement in glucose and lipid metabolism, enhancement of insulin sensitivity, increased antioxidant defense, and downregulation of lipogenic enzymes.

## Figures and Tables

**Table 1:** Compositions of the normal pellet diet (NPD) and high fat diet (HFD) fed to rats (g/kg).

Ingredients	NPD	HFD
Casein	212.0	342.0
L-cystine	3.0	3.0
Starch	439.0	172.0
Sucrose	100.0	172.0
Cellulose	50.0	50.0
Ground nut oil	—	25.0
Tallow	—	190.0
Maltodextrin	100.0	—
Soya bean oil	50.0	—
AIN salt mix	35.0	35.0
AIN vitamin mix	10.0	10.0

Total (g)	999.0	999.0

**Table 2:** Chemical compositions of aqueous extract of *Trigonella foenum-graecum* (AqE-TFG).

Compositions	AqE-TFG	
Amino acids (mg/g)	Aspartic acid	24.30
	Serine	13.70
	Glutamic acid	30.50
	Glysine	04.00
	Histidine	32.40
	Arginine	42.20
	Threonine	16.70
	Alanine	07.70
	Proline	10.70
	Cystine	27.50
	Tyrosine	08.80
	Valine	07.60
	Methionine	02.60
	Lysine	03.10
	Isoleucine	09.50
	Leucine	13.10
	Phenylalanine	10.40
	Tryptophan	04.90
Galactomannan (%; w/w)		32.5
Phenolic contents (mg/g)		23.12 ± 0.58
Flavonoid contents (mg/g)		2.55 ± 0.12

**Table 3:** Effect of aqueous extract of *Trigonella foenum-graecum* (AqE-TFG) on body weight gain, food intake, water intake, BMI, and adiposity index in high fat diet- (HFD-) induced obese rats.

Groups	Body weight gain (g)	Food intake (g/day)	Water intake (mL/day)	BMI (kg/m^2^)	Adiposity index (%)
Normal control	54.60 ± 3.57	15.64 ± 1.07	37.10 ± 0.68	4.23 ± 0.07	1.49 ± 0.04
HFD control	122.93 ± 8.03^##^	17.05 ± 0.92	40.53 ± 1.04	5.92 ± 0.27^##^	2.24 ± 0.08^##^
HFD + AqE-TFG (0.5 g/kg)	103.17 ± 5.72*	16.40 ± 0.73	38.42 ± 0.85	5.16 ± 0.15**	1.97 ± 0.03**
HFD+ AqE-TFG (1 g/kg)	90.52 ± 4.05**	14.79 ± 0.60	37.98 ± 1.21	4.89 ± 0.08**	1.80 ± 0.06**
HFD + orlistat (10 mg/kg)	83.76 ± 4.63**	15.95 ± 0.84	39.00 ± 1.36	4.72 ± 0.23**	1.74 ± 0.04**

Results are expressed as mean ± S.E.M. (*n* = 10); ^##^ *P* < 0.01 compared to normal control; *P < 0.05 and **P < 0.01 compared to HFD control.

**Table 4:** Effect of aqueous extract of *Trigonella foenum-graecum* (AqE-TFG) on blood pressure in high fat diet- (HFD-) induced obese rats.

Groups	HR (beats/min)	SBP (mm Hg)	DBP (mm Hg)	MAP (mm Hg)
Normal control	428.5 ± 14.34	126.4 ± 3.40	92.3 ± 5.04	103.67 ± 3.55
HFD control	530.7 ± 26.39^##^	153.3 ± 2.16^##^	112.8 ± 3.54^##^	126.3 ± 3.02^##^
HFD + AqE-TFG (0.5 g/kg)	492.2 ± 18.95	136.5 ± 4.94*	97.4 ± 3.60*	110.43 ± 3.58**
HFD + AqE-TFG (1 g/kg)	476.1 ± 15.32	134.9 ± 3.87*	96.2 ± 2.63*	109.10 ± 1.98**
HFD + orlistat (10 mg/kg)	464.5 ± 21.79	131.1 ± 5.63**	94.6 ± 3.35**	106.77 ± 2.40**

Results are expressed as mean ± S.E.M. (*n* = 10); ^##^ *P* < 0.01 compared to normal control; *P < 0.05 and **P < 0.01 compared to HFD control.

**Table 5:** Effect of aqueous extract of *Trigonella foenum-graecum* (AqE-TFG) on organ weight and white adipose tissue (WAT) weight in high fat diet- (HFD-) induced obese rats.

Groups	Organ's weight (g)		WAT weight (g)		
	Heart	Liver	Mesenteric	Gonadal	Retroperitoneal
Normal control	0.71 ± 0.02	7.13 ± 0.24	0.64 ± 0.03	1.72 ± 0.07	1.10 ± 0.04
HFD control	0.98 ± 0.06^##^	10.58 ± 0.46^##^	1.27 ± 0.07^##^	3.20 ± 0.15^##^	2.14 ± 0.09^##^
HFD + AqE-TFG (0.5 g/kg)	0.84 ± 0.03*	9.04 ± 0.30**	1.06 ± 0.05**	2.54 ± 0.09**	1.83 ± 0.05**
HFD + AqE-TFG (1 g/kg)	0.80 ± 0.04*	8.20 ± 0.17**	0.90 ± 0.04**	2.17 ± 0.07**	1.59 ± 0.08**
HFD + orlistat (10 mg/kg)	0.78 ± 0.02**	7.86 ± 0.22**	0.85 ± 0.03**	2.06 ± 0.10**	1.48 ± 0.07**

Results are expressed as mean ± S.E.M. (*n* = 10); ^##^ *P* < 0.01 compared to normal control; *P < 0.05 and **P < 0.01 compared to HFD control.

**Table 6:** Effect of aqueous extract of *Trigonella foenum-graecum* (AqE-TFG) on serum lipid levels in high fat diet (HFD)-induced obese rats.

Groups	TC (mg/dL)	TG (mg/dL)	HDL-C (mg/dL)	LDL-C (mg/dL)	VLDL-C (mg/dL)	AI	CRI
Normal control	99.07 ± 2.84	68.32 ± 3.56	32.65 ± 2.25	52.76 ± 4.26	13.66 ± 0.71	0.32 ± 0.03	3.22 ± 0.30
HFD control	156.36 ± 4.00^##^	148.90 ± 5.29^##^	20.19 ± 1.95^##^	106.38 ± 5.45^##^	29.78 ± 1.06^##^	0.89 ± 0.05^##^	8.70 ± 0.79^##^
HFD + AqE-TFG (0.5 g/kg)	133.77 ± 2.45**	103.47 ± 4.70**	29.84 ± 1.62**	83.24 ± 2.99**	20.69 ± 0.94**	0.54 ± 0.04**	4.68 ± 0.36**
HFD + AqE-TFG (1 g/kg)	118.54 ± 3.20**	83.15 ± 3.86**	32.91 ± 1.56**	70.20 ± 4.04**	16.43 ± 0.77**	0.43 ± 0.03**	3.85 ± 0.27**
HFD + Orlistat (10 mg/kg)	112.78 ± 4.32**	76.02 ± 4.93**	33.74 ± 2.18**	63.84 ± 5.42**	15.21 ± 0.98**	0.36 ± 0.05**	3.64 ± 0.45**

Results are expressed as mean ± S.E.M. (*n* = 10); ^##^ *P* < 0.01 compared to normal control; *P < 0.05 and **P < 0.01 compared to HFD control.

**Table 7:** Effect of aqueous extract of *Trigonella foenum-graecum* (AqE-TFG) on serum leptin, glucose, insulin, HOMA-IR, QUICKI, apo-B, and adiponectin levels in high fat diet- (HFD-) induced obese rats.

Groups	Leptin (ng/mL)	Glucose (mg/dL)	Insulin (*μ*U/mL)	HOMA-IR	QUICKI	Apo-B (mg/dL)	Adiponectin (ng/mL)
Normal control	2.49 ± 0.11	103.16 ± 2.78	45.08 ± 3.10	1.90 ± 0.12	0.274 ± 0.002	2.16 ± 0.19	6421.67 ± 2.25
HFD control	8.62 ± 0.40^##^	152.83 ± 5.22^##^	87.61 ± 6.13^##^	5.54 ± 0.48^##^	0.243 ± 0.002^##^	7.34 ± 0.48^##^	6378.49 ± 3.86^##^
HFD + AqE-TFG (0.5 g/kg)	5.87 ± 0.31**	135.66 ± 3.39*	65.42 ± 3.90**	3.67 ± 0.26**	0.254 ± 0.002*	3.80 ± 0.27**	6398.37 ± 4.40**
HFD + AqE-TFG (1 g/kg)	4.23 ± 0.26**	122.17 ± 3.84**	54.13 ± 4.36**	2.74 ± 0.23**	0.262 ± 0.004**	3.03 ± 0.21**	6409.65 ± 1.98**
HFD + orlistat (10 mg/kg)	3.58 ± 0.19**	128.03 ± 4.60**	48.33 ± 2.95**	2.57 ± 0.21**	0.265 ± 0.003**	2.68 ± 0.33**	6413.27 ± 3.52**

Results are expressed as mean ± S.E.M. (*n* = 10); ^##^ *P* < 0.01 compared to normal control; *P < 0.05 and **P < 0.01 compared to HFD control.

**Table 8:** Effect of aqueous extract of *Trigonella foenum-graecum* (AqE-TFG) on serum lipase, lactate dehydrogenase (LDH), aspartate amino transferase (AST), and alanine amino transferase (ALT) levels in high fat diet- (HFD-) induced obese rats.

Groups	Lipase (U/L)	LDH (IU/L)	AST (IU/L)	ALT (IU/L)
Normal control	671.20 ± 63.59	29.67 ± 2.81	56.70 ± 3.58	27.63 ± 2.30
HFD control	1594.61 ± 121.04^##^	65.83 ± 3.75^##^	96.23 ± 4.92^##^	59.78 ± 4.50^##^
HFD + AqE-TFG (0.5 g/kg)	1237.13 ± 102.53*	46.35 ± 4.23**	75.52 ± 5.62**	42.08 ± 4.31*
HFD + AqE-TFG (1 g/kg)	1062.32 ± 78.90**	38.22 ± 2.55**	64.88 ± 3.79**	36.45 ± 3.12**
HFD + orlistat (10 mg/kg)	920.78 ± 89.75**	34.06 ± 4.44**	61.27 ± 3.16**	33.64 ± 5.16**

Results are expressed as mean ± S.E.M. (*n* = 10); ^##^ *P* < 0.01 compared to normal control; *P < 0.05 and **P < 0.01 compared to HFD control.

**Table 9:** Effect of aqueous extract of *Trigonella foenum-graecum* (AqE-TFG) on lipogenic enzymes levels in hepatic and uterine WAT in high fat diet- (HFD-) induced obese rats.

Groups	FAS (*μ*mol/min/g tissue)		G6PD (*μ*mol/min/g tissue)	
	Liver	Uterine WAT	Liver	Uterine WAT
Normal control	0.59 ± 0.03	0.12 ± 0.01	1.02 ± 0.10	0.53 ± 0.05
HFD control	1.14 ± 0.08^##^	0.25 ± 0.02^##^	1.90 ± 0.15^##^	0.86 ± 0.12^##^
HFD + AqE-TFG (0.5 g/kg)	0.91 ± 0.05**	0.18 ± 0.01*	1.32 ± 0.12**	0.60 ± 0.04*
HFD + AqE-TFG (1 g/kg)	0.74 ± 0.03**	0.15 ± 0.02**	1.18 ± 0.08**	0.51 ± 0.07**
HFD + orlistat (10 mg/kg)	0.65 ± 0.04**	0.16 ± 0.02**	1.10 ± 0.11**	0.56 ± 0.06*

Results are expressed as mean ± S.E.M. (*n* = 10); ^##^ *P* < 0.01 compared to normal control; *P < 0.05 and **P < 0.01 compared to HFD control.

**Table 10:** Effect of aqueous extract of *Trigonella foenum-graecum* (AqE-TFG) on hepatic and cardiac thiobarbituric acid reactive substance (TBARS) and antioxidant enzymes (GSH, SOD, and CAT) levels in high fat diet- (HFD-) induced obese rats.

Groups	TBARS (nmol/mg protein)		GSH (*μ*mol of P liberated/ min/mg protein)		SOD (U/mg protein)		CAT (nmol of H_2_O_2_/min/mg protein)	
	Liver	Heart	Liver	Heart	Liver	Heart	Liver	Heart
Normal control	0.20 ± 0.01	0.17 ± 0.02	27.15 ± 1.49	22.50 ± 1.07	17.73 ± 0.62	11.62 ± 0.72	54.08 ± 3.94	46.35 ± 5.12
HFD control	0.68 ± 0.03^##^	0.52 ± 0.04^##^	9.87 ± 0.82^##^	8.36 ± 1.51^##^	10.31 ± 1.04^##^	6.55 ± 0.46^##^	24.75 ± 2.11^##^	21.80 ± 3.04^##^
HFD + AqE-TFG (0.5 g/kg)	0.36 ± 0.04**	0.29 ± 0.02**	20.46 ± 2.01**	17.05 ± 1.83*	15.94 ± 0.76**	9.96 ± 1.13*	47.31 ± 5.42**	39.62 ± 4.18**
HFD + AqE-TFG (1 g/kg)	0.31 ± 0.02**	0.25 ± 0.02**	25.30 ± 1.14**	19.85 ± 2.30**	17.23 ± 0.64**	11.28 ± 0.86**	50.82 ± 3.19**	44.20 ± 3.25**
HFD + orlistat (10 mg/kg)	0.23 ± 0.01**	0.21 ± 0.01**	23.65 ± 2.26**	21.17 ± 2.58**	18.04 ± 0.88**	11.02 ± 1.29**	52.45 ± 6.72**	45.07 ± 2.47**

Results are expressed as mean ± S.E.M. (*n* = 10); ^##^ *P* < 0.01 compared to normal control; *P < 0.05 and **P < 0.01 compared to HFD control.

## Abbreviations

(AqE-TFG): Aqueous extract of* Trigonella foenum-graecum* seeds
(HFD): High fat diet
(NPD): Normal pellet diet
(BMI): Body mass index
(WAT): White adipose tissue
(apo-B): Apolipoprotein-B
(HOMA-IR): Homeostasis model assessment for insulin resistance
(CVD): Cardiovascular disease
(AST): Aspartate amino transferase
(ALT): Alanine amino transferase
(LDH): Lactate dehydrogenase
(FAS): Fatty acid synthetase
(G6PD): Glucose-6-phosphate dehydrogenase
(TBARS): Thiobarbituric acid reactive substance
(GSH): Glutathione
(SOD): Superoxide dismutase
(CAT): Catalase
(WHO): World Health Organization
(FDA): Food and Drug Administration
(ICAR): Indian Council for Agricultural Research
(PBS): Phosphate buffer saline
(AQC): 6-Aminoquinolyl-N hydroxyl succinimidyl carbamate
(HPLC): High performance liquid chromatography
(CPCSEA): Committee for the Purpose of Control and Supervision of Experiments on Animals
(IAEC): Institutional Animal Ethics Committee
(HR): Heart rate
(SBP): Systolic blood pressure
(DBP): Diastolic blood pressure
(MAP): Mean arterial blood pressure
(TGs): Triglycerides
(TC): Total cholesterol
(HDL-cholesterol): High density lipoprotein cholesterol
(LDL-C): Low density lipoprotein cholesterol
(VLDL-C): Very low density lipoprotein cholesterol
(AI): Atherogenic index
(CRI): Coronary risk index
(ELISA): Enzyme-linked immunosorbent assay
(H & E): Hematoxylin and eosin
(ANOVA): Analysis of variance
(CSIR): Council of Scientific and Industrial Research.
